# Bacteriological assessment of urban water sources in Khamis Mushait Governorate, southwestern Saudi Arabia

**DOI:** 10.1186/1476-072X-8-16

**Published:** 2009-03-21

**Authors:** Eed L Sh AlOtaibi

**Affiliations:** 1 Geographical Information Systems and Water Resources Applications, School of Human Sciences, Geography Department, King Khalid University, PO 1183, Abha, Saudi Arabia

## Abstract

**Background:**

Urban water sources of Khamis Mushait Governorate, southwestern Saudi Arabia, were studied to assess their bacteriological characteristics and suitability for potable purposes. A cross-sectional epidemiological method was adopted to investigate the four main urban water sources (i.e. bottled, desalinated, surface, and well water). These were sampled and examined between February and June 2007.

**Results:**

A total of 95 water samples from bottled, desalinated, surface, and well water were collected randomly from the study area using different gathering and analysing techniques. The bacteriological examination of water samples included the most probable number of presumptive coliforms, faecal coliforms, and faecal streptococci (MPN/100 ml). The results showed that the total coliform count (MPN/100 ml) was not detected in any samples taken from bottled water, while it was detected in those taken from desalinated, surface, and well water: percentages were 12.9, 80.0, and 100.0, respectively. Faecal coliforms were detected in desalinated, surface, and well water, with percentages of 3.23, 60.0 and 87.88, respectively. About 6.45% of desalinated water, 53.33% of surface water, and 57.58% of well water was found positive for faecal streptococci. Colonies of coliforms were identified in different micro-organisms with various percentages.

**Conclusion:**

Water derived from traditional sources (wells) showed increases in most of the investigated bacteriological parameters, followed by surface water as compared to bottled or desalinated water. This may be attributed to the fact that well and surface water are at risk of contamination as indicated by the higher levels of most bacteriological parameters. Moreover, well water is exposed to point sources of pollution such as septic wells and domestic and farming effluents, as well as to soil with a high humus content. The lower bacteriological characteristics in samples from bottled water indicate that it is satisfactory for human drinking purposes. Contamination of desalinated water that is the main urban water source may occur during transportation from the desalination plant or in the house reservoir of the consumer. Improving and expanding the existing water treatment and sanitation systems is more likely to provide safe and sustainable sources of water over the long term. Strict hygienic measures should be applied to improve water quality and to avoid deleterious effects on public health, by using periodical monitoring programmes to detect sewage pollution running over local hydrological networks and valleys.

## Background

High-quality water sources may be required only for drinking purposes, while the quality of water for other domestic uses can be quite variable. Therefore, water polluted to only a certain extent can be considered pure [1]. With an increasing urban population density of the study area, the scarcity and pollution of surface water poses a serious problem for urban drinking water supplies of metropolitan areas [2-7]. Consequently, water resources are a key factor, particularly for planning a sustainable socioeconomic development [8,9]. Bottled water, however, is being widely consumed because it contains fewer impurities. Therefore, it can also be beneficial to detect deterioration in the quality of water resources and to facilitate appropriate and timely corrective actions with a minimal negative impact on public health [10-14].

For the last three decades, many countries in arid and semi-arid regions have depended heavily on the desalination of seawater to meet their growing needs. Saudi Arabia is considered one of the biggest efficient producers of freshwater by desalination, with an installed capacity of more than 1000 million USGPD, accounting for 24.4%> of the world's desalinated water production [15,16]. In the case of Saudi Arabia, surface water sources (i.e. dams, lakes, and open water reservoirs) are considered to be very limited resources and are exploited for almost every use. They are also exposed to urban wastewater disposal from both wastewater stations (that has not reached secured stages 3–4 in most of the Saudi wastewater stations 70%, causing an expected environmental pollution especially around metropolitan areas), which has made the surface water resources highly polluted, especially in parched valleys. The frequent outbreaks of waterborne diseases are the result of a direct discharge of untreated or partially treated domestic sewage water sources located beside local gutters [1,17,4].

Groundwater is still and will continue to be the main source of safe and reliable drinking water, especially in rural areas in Saudi Arabia. Water taken from such sources (different types of shallow and deep wells) is often of better quality than surface water or other open water sources if the soil is fine-grained and its bedrocks do not have cracks, crevices, and bedding plants, which permit the free passage of polluted water especially within metropolitan zones [7,30,37,7,20]. It is often assumed that natural, uncontaminated water from deep wells is clean and healthy, and this is usually true with regard to bacteriological composition [21]. However, bacterial pollution of water sources may occur and is mostly derived from watershed corrosion as well as drainage from sewage, swamps, or soil with a high humus content. This type of hazard exists particularly in limestone areas where underground chambers or fissures may permit water to flow in the freely moving streams without substantial filtrations. Such suspected water sources cannot be used without caution for human drinking purposes because of the inherent health risks [22,16,17,24,4-7,11].

The major interest of public health authorities in developing quality standards for urban water uses is focused on the recognition, enumeration, identification, and assessment of microorganisms related to waterborne diseases that are considered indicators of microbiological parameters [25,17,5,7,11,26]. These indicators are of great importance to assess the microbial condition of the examined water sources [27]. Moreover, the bio-indicator of faecal pollution is a non-pathogenic microorganism, whose detection suggests the presence of enteric pathogens. Usually, coliforms, faecal coliforms, and faecal streptococci are recognised as the main indicators of microorganisms in water sources [26,9]. These indicators are of significance to assess the microbial condition of the water supply [28]. Microorganisms as an indicator of faecal pollution should satisfy several criteria [29,20]. For instance, they should be present in faeces in greater numbers and have more resistance than any pathogen to the stresses of an aquatic environment [30,31,17,4,26]. The evaluation of total coliforms may sometimes give erroneous information regarding faecal contamination [32,17,4,11,26,12].

The main objective of this study was to assess the bacteriological water quality and its geospatial relations of the four major urban water sources in the study area (bottled, desalinated, surface, and well water) which have been the focus of the community [23,5-7]. An attempt was also made to identify the coliforms isolated from the examined water samples (Table 1). The findings may be considered as a basis for water health policy decisions at different administrative levels in the study area.

**Table 1:** Frequency distribution of the isolated coliform groups in water samples from Khamis Mushait Governorate, southwestern Saudi Arabia (February-June 2007)

	**Desalinated**		**Surface water**		**Wells**		**TOTAL**	
**Microorganism**	**No.**	**%**	**No.**	**%**	**No.**	**%**	**No.**	**%**
***Escherichia coli***	2	20.00	11	24.44	10	16.95	23	20.18
***Klebsiella species:***								
*Kl. Pneumoniae*	2	20.00	3	6.67	8	13.56	13	11.40
*Kl. Oxytoca*	1	10.00	2	4.44	4	6.78	7	6.14

***Enterobacter species:***								
*E. cloacae*	1	10.00	8	17.78	11	18.64	20	17.54
*E. aerogens*	N|A	N|A	3	6.67	3	5.08	6	5.26
*E. agglomerans*	N|A	N|A	4	8.88	3	5.08	7	6.14
*E. gergoviae*	N|A	N|A	1	2.22	2	3.89	3	2.63

***Citrobacter species:***								
*C. freundii*	1	10.00	3	6.67	6	10.16	10	8.77
*C. diversus*	N|A	N|A	5	11.11	3	5.08	8	7.02
***Proteus species:***								

*P. vulgaris*	2	20.00	4	8.88	6	10.16	12	10.52
*P. mirabilis*	1	10.00	1	2.22	3	5.08	5	4.39

**TOTAL**	10	100.00	45	100.00	59	100.00	114	100.00

N|A = Not detected.

## Results and discussion

**Figure 1:**
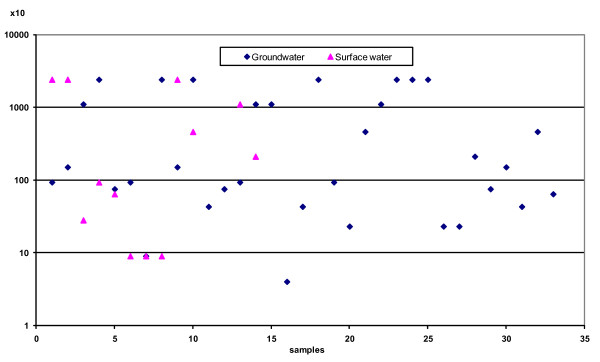
**Total coliforms in both groundwater and surface water**.

Data recorded in Figure 1 indicated that total coliforms were not detected in any sample taken from bottled water. In the desalinated water, surface water, and well water, total coliforms were detected with percentages of 12.9, 80, and 100.0, respectively. However, log counts of total coliform bacteria (MPN/100 ml) in desalinated, surface, and well water were 0.0–1.60, 0.0-≥ 4.38, and 1.60-≥4.38, respectively. The log mean values were 3.79 ± 3.40 and 3.86 ± 3.22 (MPN/100 ml) in samples taken from surface and well water, respectively. In previous studies, total coliform bacteria were detected in different water sources with various mean values and percentages [28,31,23,33,34]. There was no significant correlation in the level of total coliforms between well and surface water. As previously cited, total coliform counts must not be detected in any 100 ml water samples [35,17,24,11]. Therefore, results of total coliforms recorded in the present study showed that all examined samples from wells (100.00%) and most surface water (80.00%) exceeded the guideline values recommended in accordance with international standards [3,17,24].

The most common group of indicator organisms used in water quality monitoring are coliforms. These organisms are representative of bacteria normally present in the intestinal tract of mammals including human, so they provide a general, albeit adequate, index of faecal contamination of drinking water [36,24,26,38]. Moreover, the presence of coliforms in drinking water could also indicate a breakdown of the treatment process [28]. The transportation of desalinated water by tanker does not contribute significantly. Such contamination obviously occurs during storage in the house reservoir (earth) and is possibly implicated, at least partly, in the increased prevalence of diarrhoea [23].

**Figure 2:**
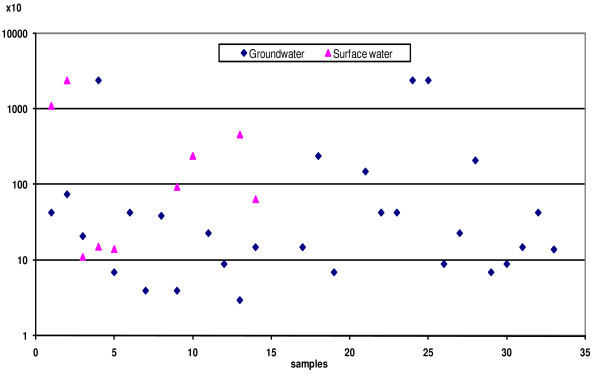
**Faecal coliforms in both groundwater and surface water**.

From the results recorded in Figure 2, it is evident that faecal coliforms were not detected in any samples taken from bottled water, while from desalinated water, only one out of 31 (3.23%) samples was found positive for faecal coliforms. However, 9 out of 15 (60.0%) and 29 out of 33 (87.88%) specimens were found positive for faecal coliforms in samples taken from surface and well water, respectively. The log counts of faecal coliforms (MPN/100 ml) ranged from 0.0 to 1.6; 0.0 to ≥ 4.38 and 0.0 to ≥ 4.38 in desalinated, surface, and well water, respectively. Logarithmic mean values (MPN/100 ml) were 3.47 ± 3.23 and 3.40 ± 3.08 in surface and well water, respectively. There was no significant correlation in the level of faecal coliforms between well and surface water. These results indicated that most samples taken from wells (87.88%) and surface water (60.00%) had higher faecal coliforms with respect to the international guideline value, in which drinking water must be free from faecal coliforms [22,17,24,11,26,9]. Different coliform counts were previously recorded in groundwater samples [28,31,23,39,40].

Indicators such as faecal coliforms are not the best, because their effectiveness will be minimised in geographical zones when the temperature is high [41,26,42]. However, well water is at risk of contamination, as indicated by the presence of faecal coliforms [43,24,5,7,11,44,20].

**Figure 3:**
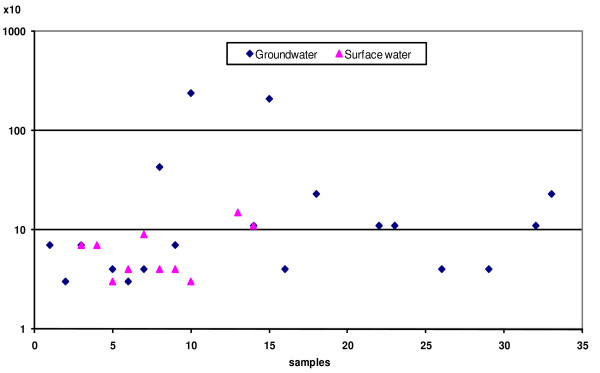
**Faecal streptococci in both groundwater and surface water**.

It is evident from Figure 3 that faecal streptococci were not detected in any samples taken from bottled water. Two out of 31 (6.45%) desalinated water samples, 8 out of 15 (53.33%) surface water samples, and 19 out of 33 (57.58%) well water samples were found positive for faecal streptococci. Logarithmic range values of faecal streptococci (MPN/100 ml), however, were 0.0–1.6, 0.0–2.18, and 0.00–3.38 in samples taken from desalinated, surface, and well water, respectively. The log mean values of faecal streptococci (MPN/100 ml) were 1.65 ± 1.07 and 2.28 ± 1.97 in surface and desalinated water. There was a significant correlation at p = 0.05 in the level of faecal streptococci between surface and well water. With regard to international guideline values, in which water must be free from faecal streptococci, 6.45% of the desalinated water, 53.33% of the surface water, and 57.58% of the well water was considered to be unfit for drinking purposes. Faecal streptococci were previously isolated with various frequencies [28,31,33]. Enterococcus species were formerly classified in the genus streptococci. They are primarily commensurate with residence in the intestine, though some also cause gastroenteritis, nosocomial infection, endocarditis, intra-abdominal infection, surgical wound infection, and urinary tract infections [45,46,19,17,24,11].

As regards the bacteriological examination of water sources carried out in this study, high total coliforms, faecal coliforms, and faecal streptococci in surface and most well water are considered an indication of recent faecal pollution from human or animal excreta, which may reflect the possibility of potential health hazards [42]. The primary risk of consuming untreated water is the transmission of communicable diseases by pathogenic organisms. Those present in aquatic environments can be of natural origin or may be discharged by humans and other warm-blooded animals. However, the water, which is not suitable for drinking, may be usable for irrigation or for other domestic purposes. Thus, it can be seen that each use of water imposes its own limits on the degree of pollution that can be considered acceptable [1,17,24]. Drinking only from desalinated water sources was associated with diarrhoea as compared with drinking only from bottled water or from any other sources [23]. Water from the valleys and wells of the study area was grossly polluted and was used regularly for purposes other than drinking [23,5-7].

The coliform group comprises strains of the four genera of the intestinal group: Escherichia, Enterobacter, Klebsiella, and Citrobacter. The number of Escherichia and Enterobacter remains much higher in the intestine than do the remaining two [1,26,9].

The frequency distribution of the different microorganisms isolated from the examined samples is given in Table 1. A total of 114 isolated bacteria included 10 from desalinated water, 45 from surface water, and 59 from wells. These were typed to be 23 Escherichia coli (E. coli), 13 Klebsiella pneumonia, 7 Klebsiella oxytoca, 20 Enterobacter cloacae, 6 Eenterobacter aerogens, 7 Eenterobacter agglomerans, 3 Enterobacter gergoviae, 10 Citrobacter freundii, 8 Citrobacter diversus, 12 Proteus vulgaris, and 5 Proteus mirabilis, with percentages of 20.18, 11.40, 6.14, 17.54, 5.26, 6.14, 2.63, 8.77, 7.02, 10.52, and 4.39, respectively. Most of these bacterial species had been previously isolated from different water sources, although their percentages varied [47,27,31,40,11].

It is clear that out of all possibilities, E. coli can best fulfil conditions possible to act as an ideal indicator of faecal pollution. These organisms survive longer in water than most pathogens, and thus can detect recent as well as earlier pollution. In terms of public health significance, E. coli has frequently been reported to be the causative agent of traveller's diarrhoea, urinary tract infection, haemorrhagic colitis, and haemolytic uraemia syndrome. Moreover, Klebsiella pneumonia is associated with pneumonia and upper respiratory tract infection. However, Enterobacter and Citrobacter species have also been previously reported as causes of cystitis, enteritis, pneumonia, diarrhoea, and food poisoning [48,17,24,11]. Proteus species are apparently of epidemiological importance in summer diarrhoea in infants and in food-borne outbreaks. Proteus vulgaris in association with other bacteria has been reported to be the causative agent of cystitis and pyelitis [48,25,17,24,11].

Based on the above assessments, although bottled water may be of good quality in the Khamis Mushait Governorate urban area, the public supply of both desalinated water distributed via an urban water network system to areas of city quarters and conventional water sources such as wells and surface water cannot be ignored by local water authorities. They should consider a proper regular monitoring programme (i.e. wells and surface water microbial source tracking system) to determine the primary sources of contamination, their contribution, health threat, and geographic distribution. In addition, they ought to make recommendations and to develop appropriate control measures to avoid any sudden public health risk from such a vital water source [23,11].

## Conclusion

Water derived from traditional sources (wells) showed increases in most of the investigated bacteriological parameters, followed by surface water as compared to bottled or desalinated water. This may be highly attributed to the fact that well and surface water of Khamis Mushait Governorate is at risk of contamination as indicated by the higher levels of most bacteriological parameters. Moreover, well water is exposed to point sources of pollution such as septic wells and domestic and farming effluents as well as to soil with a high humus content [4,11]. The lower bacteriological characteristics in samples of bottled water indicate that it is satisfactory for human drinking purposes. Nevertheless, contamination of desalinated water may occur during its transportation from the desalination plant to the consumer or during storage in a house reservoir. Improving and expanding the existing water treatment and sanitation systems is more likely to provide good, safe, and sustainable sources of water in the long term. Strict hygienic measures should be applied to improve water quality and to avoid deleterious effects on public health [3,6,11]. This could be achieved by upgrading current sewage stations (i.e. to deal with stages 3 and 4) and adopting a periodical monitoring programmes to detect sewage pollution in water supplies, [23,5-7,14] thus eliminating the possibility that disease may be transmitted by their use or during their running through the local hydrological network and valleys as have been noticed via satellite digital mapping of the study area [17,4,6].

## Methods

### Study area, design, samples, and materials

#### The study area

The study was conducted in an urban zone of Khamis Mushait Governorate (about 43 km × 25 km centred at 18.3° N, 42.8° E [42], with a population of 497,000 [2007]), which covers about 1075 km^2^, with an elevation ranging from about 982 to 1946 m (mean 1464 m) above sea level, an average annual rainfall of 355 mm (range 160–450 mm), it has a two short rainy seasons, 70% of which occurs in March and May (ranges between 40–55 mm) and August and September (ranges between 36–62 mm) with about 300 mm/y and average minimum and maximum temperatures of 19.3 C and 29.70 C, respectively [17,5-7].

#### Design

In this study, a cross-sectional epidemiological method was used to assess representative samples of the four main urban water sources (i.e. bottled, desalinated, surface, and well water; see Table 1) in Khamis Mushait Governorate, southwestern Saudi Arabia. These representative samples were examined between February and June 2007 to assess their bacteriological characteristics and suitability for potable purposes. Using a simple random sampling technique, a total of 95 drinking water samples were collected from bottled water, desalinated water, surface water, and groundwater (wells of different types).

#### Sampling and materials

Simple random sampling was the method chosen for this study. Geographical settings of both the surface water and wells were determined in advance via a digital satellite mapping processing technique (Erdas Map sheet, v.9 and Global Mapper Software, LLC v. 10) by using the Google Earth digital mapping engine (a paid copy of Google Earth pro™) to obtain an overview, to ease virtual navigation, and to refine the micro-geographic data when mapping the Khamis Mushait administrative area [49,5-7]. (Satellite images are aerial photographs and do not represent real time; they have an average high resolution age of several years and a spatial resolution of 25 m per pixel or even higher [15 m] in some areas) [42].

The network sampling method offered options that may have been more efficient for this study than classical sampling [50]. It employed good local knowledge, including of streets, in determining targeted water groups, their geographic distribution, and boundaries using Google Earth digital maps as a powerful platform in improving micro-sampling, processing, field manipulations and operations, tracking, allocation, and high-quality map creation. All of these elements supported the training of the research sampling team and helped in understanding the spatiotemporal relationship and geographic patterns between all entities. Composite maps of different types (i.e. hand drawn maps) were also used efficiently by the researcher and the field support team for the disk and ground phases.

For bottled water, sixteen brands (known to the local community) consisting of spring and purified bottled water types were purchased from different local supermarkets within Khamis Mushait Governorate and sampled. For desalinated water, 31 water samples were obtained from *Ashiab *(i.e. distributing points for the Khamis Mushait Governorate water desalination station), using the simple random sampling technique, from water trailers, houses, urban water networks, fish markets, and slaughterhouses. For surface water, 15 specimens were collected from the selected sites, the *Tandaha *dam reservoir and valleys around Khamis Mushait Governorate, using the simple random sampling technique. From wells, 33 water samples were also selected from the chosen geo-sites of different types of wells located around the study area, using the simple sampling technique. Planning of both the surface and groundwater samples was carried out, and the specimens were assessed using spatial techniques (i.e. network method) for the digital satellite map of Khamis Mushait Governorate, using the Google Earth mapping engine [42].

Samples from each brand of bottled water were kept in a screw capped 1.5-litre plastic container. Samples from desalinated, surface, and well water were collected under completely sterile conditions and placed in sterile, screw capped, 150-ml plastic bottles, taking into consideration the standard methods of both gathering and handling water samples. All specimens of desalinated, surface, and well water were sampled and dispatched daily, with a minimum delay, in an FWD Car (provided by King Khalid University to the author and his trained sampling team [E AlOtaibi, MSA Zaki A Ghorm, and N Alshahrani]) to the Medical Laboratory Technology Department, Khamis Mushait Community College. Most water quality constituents were determined within 2–6 hours of collection [3].

The bacteriological examination of water samples includes Most Probable Number (MPN) of presumptive coliforms, faecal coliforms, and faecal streptococci (MPN/100 ml water) using the Multiple Tube Fermentation Technique [3,26]. Suspected colonies of coliform groups were also identified on the basis of morphological, cultural, and biochemical characteristics [51,9]. Significant differences between each two means were evaluated using SPSS-PC Version 11 of the Student-T-Test [52].

### Quality assurance procedures

#### Sampling strategy and design

##### Disk preparation phase

○ Adoption of a two-stage sampling scheme.

○ Careful planning and choice of representative sampling groups and sites according to the adopted network sampling technique, and determining certain criteria such as control sites where major sampling groups exist (i.e. surface water points, valleys, and wells), impact sites where contamination is expected, such as polygons, and outlets (e.g. treated water discharges site) to maximise understanding the quality of urban water sources, and with the least risk of missing the correct representative sampling groups and sites.

○ Attention paid to ensure inclusion in the sampling frame of all groups and locations (sites, roads, venues, and so on) via screening, browsing, and delineation from a satellite digital map of the Khamis Mushait Governorate zone, because local pre-knowledge was preferred with regard to accessibility, safety, and permission.

○ Approximation of the number of the target study population in each group and sampling location.

○ Determination of the proportional allocation of samples between different groups and locations.

○ Training of interviewers\sample collectors to follow and to use the sampling strategy and procedures.

○ Implementation of ways to boost participation rates in the screening and core interviews and sample collection.

○ Planning of logistic needs of timing, gathering, handling samples, and laboratory.

○ Producing sampling cards to be completed to record observations at scene (sample ID and data/information: date, time, temperature, group, locality, problems in the area, sketch map).

○ Selection of an appropriate major sampling method [52] (i.e. simple random sample; network sampling).

○ Planning of pilot visits to samples of each group in the field to review strategy.

##### During sampling phase

▪ Use simple random selection procedures when feasible to select representative samples of each location for each group.

▪ Gather water specimens from each sampled location with a probability proportional to the estimated total of the target population.

▪ Interview all eligible persons with regard to this site (as auxiliary data).

▪ Collect auxiliary data (on sampled site, it may affect the probability of selection)

▪ Transport and store samples away from sunlight or extreme heat.

##### During analysis phase

○ Perform standard analysis procedures.

○ Compare results and findings of each sample within each group with their auxiliary data and other associated characteristics written on sample card to verify and to make sure it belongs to the same group and sampled location; assess reasons for refusal if there are any and determine whether refusal is associated with selection biases or just handling, and report immediately.

○ Assess representativeness of the selected samples by comparing the data with other data.

○ Incorporate weights into the analyses to reflect unequal probabilities of selection, incomplete sampling frame, and rates of refusal samples.

○ Assess the need to use statistical programmes that incorporate the design effect of such a cross-sectional study.

○ Compare findings relating to the collected samples with expected results of the groups.

## Competing interests

The author declares that they have no competing interests.

## About the author

Dean of Admission and Registration, Najran University; Supervisor of ITC, eLearning and Training Centre; Academic Accreditation Centre, 2006-present, Najran University, Post Office Box: 1988, 11001, Najran, Saudi Arabia.
